# Non-bacterial Thrombotic Endocarditis in Lung Cancer: A Systematic Review

**DOI:** 10.2174/011573403X343187250117062341

**Published:** 2025-02-11

**Authors:** Maikel Kamel, Fahad Hussain, Christian Leung, Awais Paracha, Pranav Sathe, Ajay Jassal, Mahalia Huba, Umar Durrani, Nadim Ammari, Robert S. Copeland-Halperin, Nagashree Seetharamu

**Affiliations:** 1 Department of Medicine, North Shore University Hospital, Manhasset, NY, 11030, United States;; 2 Department of Medicine, Medical University of South Carolina, Charleston, SC, 29425, United States;; 3 Department of Medicine, School of Medicine, Saint Louis University, Saint Louis, MO, 63103, United States

**Keywords:** Non-bacterial thrombotic endocarditis (NBTE), malignancy, lung cancer, hypercoagulable state, anticoagulation, valvular vegetations

## Abstract

**Introduction:**

Non-bacterial Thrombotic Endocarditis (NBTE) is a rare condition characterized by aseptic vegetations of the heart valves, predisposing to valvular dysfunction and end-organ infarction. Lung Cancer (LC) is amongst the most common malignancies associated with NBTE.

**Methods:**

PubMed/MEDLINE was searched from database inception until January 2024, pairing Non-bacterial Thrombotic Endocarditis (NBTE) and related terms with “Lung Cancer (LC)”. Reports were included if patients had both NBTE and lung cancer. The risk of bias was assessed using Mixed Methods Analysis Testing (MMAT).

**Results and Discussion:**

32 patients with an average age of 59y +/- 11.6 were included from 31 peer-reviewed publications, with significant findings as below:

• The majority (47%) of patients were admitted with stroke.

• The most commonly affected valve was aortic (51%), followed by mitral (43%), and tricuspid (5%).

• At diagnosis of NBTE, 86% of patients had stage IV cancer.

• Multi-organ infarct was common (61%), with the brain most often affected (40%).

• Treatment of NBTE included antibiotics (86%), anticoagulation (50%), and cardiac surgery (6%).

• Treatment of LC included traditional chemotherapy (30.7%), radiation (16%), tyrosine kinase inhibitors (11.5%), lobectomy (6%), and immunotherapy (3.8%).

• Overall mortality rate was 77%.

• Mortality rate was 38% in patients treated with chemotherapy and 91% in patients who did not receive chemotherapy.

• Mortality rate stratified by anticoagulant: unfractionated heparin (85.7%), DOAC (75%), and LMWH (20%).

**Conclusion:**

High clinical suspicion for NBTE in patients presenting with LC and thromboembolic phenomena can lead to changes in treatment and improved clinical outcomes.

## 1 INTRODUCTION

Non-bacterial Thrombotic Endocarditis (NBTE), also known as Marantic Endocarditis, is a medical condition characterized by sterile vegetations on cardiac valves in the absence of a bacterial infection.

Notable predisposing conditions include hypercoagulable conditions such as malignancy, chronic inflammatory conditions, rheumatologic and autoimmune disorders, including Systemic Lupus Erythematosus (SLE), Antiphospholipid Syndrome (APLS), rheumatic heart disease, and HIV [1].

NBTE is considered rare, with a study examining adult autopsy reports among cases of endocarditis finding an incidence between 0.9 and 1.6% [2]. Malignancy is the most common associated condition, present in 75-80% of cases. NBTE has been reported in every age group, most commonly in patients between the fourth and eighth decades of life with no sex predilection [3].

The pathophysiology of NBTE in malignancy is thought to be mediated by valvular endothelial cell injury in an underlying hypercoagulable state. Specifically, antigen-presenting cells like macrophages interact with malignant cells to release cytokines, including tumor necrosis factor and interleukins, which damage the endothelium [4]. The valvular endothelial injury results in local aggregation of platelets, migration of inflammatory mononuclear cells, and deposition of immune complexes to form a thrombus interwoven with fibrin [5]. Vegetations are frequently left-sided, with two-thirds of cases involving the mitral valve, up to a quarter involving the aortic, and, less commonly, both valves [3]. Cases with all four valves affected by NBTE have also been reported [6].

The current understanding of the epidemiology and prognosis of cancer-associated NBTEs is derived from case reports and series. A recent meta-analysis of cancer-associated NBTEs showed Lung Cancer (LC) to be the most associated cancer, followed by pancreatic cancer and gynecologic malignancies [7]. The meta-analysis also showed that cancer-associated NBTE presented more commonly at an advanced stage, with LC being the most common [7]. The predisposition of NBTE towards LC was attributed to the higher hypercoagulability associated with this cancer [8].

In general, patients with NBTE are asymptomatic until embolization occurs, and they typically present with systemic emboli rather than symptoms of heart failure or valvular dysfunction [2]. In one series, stroke was the presenting complaint in 60 percent of cases [9, 10]. Common sites of embolization include the spleen, kidney, skin, and extremities which may present as flank pain, hematuria, rash, and acute limb or digit ischemia.

Currently, no laboratory tests or procedures have been validated to help diagnose pre-clinical NBTE, nor are there specific tests that distinguish NBTE from infective endocarditis. NBTE, therefore, is a disease of exclusion after an appropriate workup has ruled out an infectious cause. Once NBTE is identified, a thorough workup for malignancy, SLE, APLS, and Disseminated Intravascular Coagulation (DIC) should be conducted. Transthoracic E (TTE) and/or Transesophageal Echocardiography (TEE) are used for imaging vegetations, with TEE being more sensitive, particularly for smaller (less than 5 millimeters) lesions, or those located on the tricuspid or pulmonic valve [11]. Cardiac Magnetic Resonance Imaging (cMRI) can be helpful for distinguishing vegetation from neoplasm or thrombus [12].

Management of NBTE involves systemic anticoagulation and treatment of the underlying condition. Anticoagulation (AC) is a mainstay of NBTE, provided there is no contraindication, such as hemorrhagic stroke. Presently, there is no head-to-head prospective trial to determine which type of anticoagulation is the safest and most efficacious in NBTE [13-15]. Anticoagulation should be continued indefinitely, whenever possible. Surgery for NBTE has not been evaluated specifically in prospective studies, with criteria for surgical management extrapolated from Infective Endocarditis (IE) [16].

The prognosis of NBTE is historically grim due to its association with advanced malignancy [9]. However, it should be noted that the true prevalence of subclinical or asymptomatic NBTE with LC is unknown, and the prognosis may differ from symptomatic NBTE. Additionally, it is also unknown if the poor prognosis associated with symptomatic NBTE is driven by the underlying malignancy rather than just the NBTE.

As mentioned above, the literature on NBTE in LC is limited to case reports and series. The data these studies provide is valuable but has not been studied in aggregate to ascertain characteristics of NBTE, specifically in relation to LC, which we hypothesized might present differently than NBTE in general malignancy. In this review, we aim to elucidate trends in epidemiology, clinical presentation, and outcomes of NBTE in patients with LC.

## 2 METHODS

### 2.1 Literature Search

A comprehensive literature search was conducted using PubMed/MEDLINE, covering articles from the database's inception to January 2024. Primary search terms including, “Non-bacterial Thrombotic Endocarditis (NBTE)”, “marantic endocarditis”, “sterile endocarditis”, and “libman-sacks endocarditis”, were paired in all possible combinations with the terms “lung cancer”, “adenocarcinoma of the lung”, “non-small cell lung cancer”, “lung squamous cell carcinoma”, “lung large cell tumor”, and “lung neuroendocrine tumors”. Filters were applied to the search results to include only full-text articles, studies published in English, and studies including human subjects.

### 2.2 Study Selection

Two independent reviewers (F.H. and A.P.) assessed the eligibility of relevant papers according to the inclusion and exclusion criteria. The study inclusion criteria were broad and included all case reports and case series discussing NBTE and LC. The searches had filters applied to include only full-text articles, articles published in English, and studies involving humans. Studies were excluded if they did not report NBTE and LC or were duplicates of studies found in previous searches. To avoid the risk of individual biases, a consensus approach was employed to evaluate all selected studies. Each record was independently assessed by both reviewers, and no automation tools were used in evaluating studies for inclusion.

### 2.3 Data Selection

**Fig. (1):**
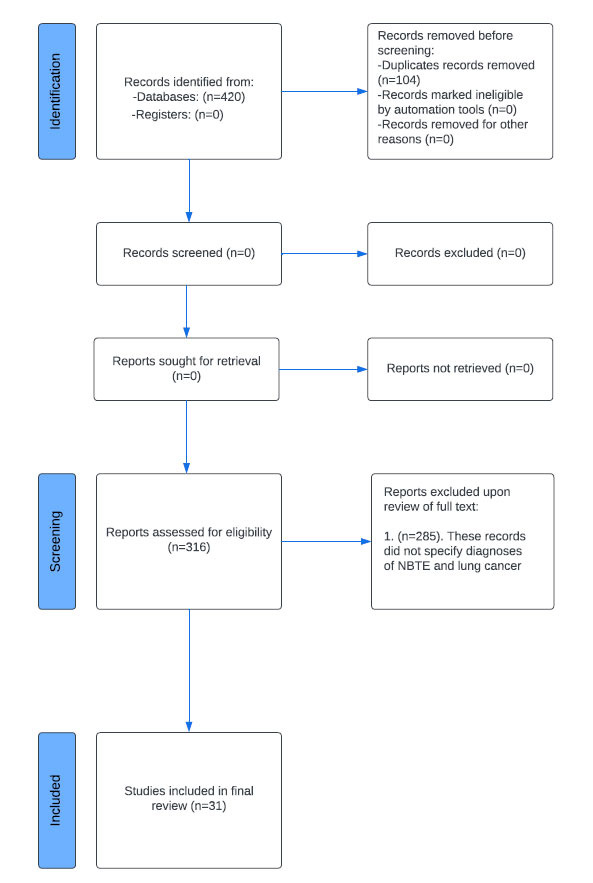
Study selection process from initial search to reports included in the final review.

Outcomes of interest included presentation, diagnosis, work-up, treatment, and mortality. The following data points were collected from each study: patient age, sex, past medical history, presenting symptoms, presenting diagnosis, presence or absence of organ infarcts, location of organ infarcts if present, involved heart valves, presence or absence of metastases, the chronology of LC or NBTE diagnosis, time from presentation to diagnosis of NBTE and LC, examination findings, laboratory data, blood culture results, laboratory markers of hypercoagulable state, echocardiogram findings, CT imaging, stage and histology of LC, use of anticoagulants, type of anticoagulation employed, cancer treatment, patient outcomes, and other diagnostic testing. Results for each data point were collected from all studies. One author (M.K.) collected the data from the selected studies and these data were checked for accuracy by multiple members of the team. Descriptive analysis was conducted to identify trends in the data. Instances of absent data were omitted from the denominator for calculating percentages for that particular point. Two reviewers (U.D. and F.H.) conducted a risk of bias analysis to assess the quality of studies included using the Mixed Methods Appraisal Tool (MMAT). No automation tools were used in the data selection process. The results of individual studies are displayed visually in the “patient characteristics” and “diagnostic work-up” tables (Fig. **1**).

## 3 RESULTS

### 3.1 Description of Patients and Neoplasms

This research identified 31 individual case reports describing 32 unique patients with LC and NBTE. The sample included 17 males (54%) and 15 females (46%) with a median age of 62.5yr (IQR +/- 12) (Table **1**).

### 3.2 Presenting Symptoms

The most prevalent initial presenting symptoms were neurologic: slurred speech (n=6) [15-20], altered mental status (n=6) [17, 21-26], visual disturbances (n=5) [27-31], weakness (n= 4) [16, 19, 30, 31], headache (n=3) [27, 29, 31], and gait abnormalities (n=2) [31-34]. Other common presentations included chest pain (n=8) [17, 26, 27, 34-38], dyspnea (n=6) [32, 37-41], fever (n=6) [25, 26, 35, 36, 42, 43], extremity pain/swelling (n=4) [34, 38, 40, 44], cough (n=2) [35], nausea/vomiting (n=2) [29, 45], back pain (n=1) [46], and hemoptysis (n=1) [35]. Two reports did not specify initial presenting symptoms [16, 47].

### 3.3 Past Medical History

Of the 32 cases, past medical history was described for 24 patients. The most common past medical history included current or former tobacco smoking (n=12) [16, 21, 27, 31-34, 38, 40, 41, 43], hypertension (n=4) [32, 35, 45, 46], hyperlipidemia (n=3) [32, 33, 35], deep venous thrombosis/pulmonary embolism (n=2) [21, 41], chronic obstructive lung disease (n=2) [33, 45], and coronary artery disease (n=2) [29, 33].

### 3.4 Diagnostic Presentation

The most common initial admission diagnosis was acute Cerebrovascular Accident (CVA) at 46.9% (n=15) [16, 18, 21, 24, 25, 27-32, 34, 37, 39, 41], followed by Pulmonary Embolism (PE) 18.8% (n=6) [24, 35, 37, 40, 41, 47], new lung mass/cancer 12.5% (n=4) [17, 36, 39, 44], endocarditis 6.3% (n=2) [42, 46], Disseminated Intravascular Coagulation (DIC) 6.3% (n=2) [16, 33], pneumonia 6.3% (n=2) [32, 43], cardiac arrest 3.1% (n=1) [26], psychosis 3.1% (n=1) [23], and pericardial effusion 3.1% (n=1) [44].

### 3.5 Diagnostic Work-up

Cultures of blood were negative for organisms in 100% of the 28 cases. Transthoracic (TTE) or Transesophageal Echocardiogram (TEE) was performed in 26 patients. Of these, valvular vegetation was identified in 100% of patients [16, 17, 22-25, 27-29, 31-35, 37, 39-46]. The 6 patients who did not have TTE or TEE had an autopsy performed. Of these 6 patients, the autopsy revealed one or more valvular vegetation in 100% of patients [18, 21, 26, 30, 36, 38]. Hypercoagulability workup was performed in 7 patients [16, 27, 29, 33, 40, 42], with 4 found to have some form of coagulopathy [16, 29, 32]. Among 28 patients, thrombocytopenia was reported in 5 patients [16, 23, 42, 44] and D-dimer was elevated in 5 patients [25, 36, 42, 44]. Inflammatory markers (erythrocyte sedimentation rate and C-reactive protein) were abnormal in 10 patients [16, 22, 25, 33-36, 42, 44, 46] (Table **2**).

CT imaging of the chest was performed in 26 of 28 patients. Among these, CT was highly suspicious for malignancy (*i.e.,* new mass, spiculated lesion, heterogenous pleural effusion, mediastinal/hilar lymph node involvement) in 92.3% (n=24) [16, 17, 22, 25-27, 29, 30, 32-36, 39-49]. Transbronchial biopsy or post-mortem examination revealed adenocarcinoma in 67.7% (n=21) [16, 17, 22-29, 32-37, 40, 42-44, 49], followed by adenosquamous carcinoma 9.7% (n=3) [21, 33, 47], large cell bronchogenic carcinoma 9.7% (n=3) [31, 39, 41], small cell carcinoma 6.5% (n=2) [45, 46], squamous cell carcinoma 3.2% (n=1) [30], and epithelial carcinoma 3.2% (n=1) [18].

Of the 32 patients, 28 had reported infarcts, with 60.7% (n=17) of these experiencing multiorgan infarction [16, 21, 23-27, 31, 32, 34, 35, 38, 41-43, 47]. There were 58 organ infarcts in total. The most common organ affected was the brain comprising 39.6% of infarcts (n=23) [16-18, 21, 23-30, 32, 34, 35, 37, 40-43, 47], followed by spleen 20.7% (n=12) [16, 21, 23, 25-27, 31, 33, 39, 41-43], kidney 19.0% (n=11) [16, 21, 23, 25, 26, 31, 33, 38, 39, 41-43], lung 10.3% (n=6) [16, 23, 31, 35, 38, 43], heart 8.6% (n=5) [22, 24, 31, 38, 45], and eye 1.7% (n=1) [32]. 43.8% of patients (n=14) had another indication of hypercoagulability (*i.e.,* DVT, PE, DIC) during or prior to admission [16, 21, 22, 24, 33, 35, 36, 38, 40-44, 48].

### 3.6 Valvular Localization

A total of 37 valves in 32 patients were affected. The aortic valve was most commonly affected 51.4% (n=19) [16, 18, 21-23, 27-31, 34-37, 39, 40, 42, 44], followed by mitral 43.2% (n=16) [16, 17, 21, 24-26, 32, 33, 38, 41, 43-47, 49] and tricuspid 5.4% (n=2) [16, 33]. Four patients had multi-valve involvement. These combinations included aortic-mitral (n=2) [21, 44], tricuspid-mitral (n=1) [33], and tricuspid-mitral-aortic (n=1) [16].

### 3.7 Time to Diagnosis of NBTE/Lung Cancer

In 29 cases with a specified timeline, LC was predominantly diagnosed before NBTE (n=17) [16, 17, 23, 24, 28, 30, 33, 34, 36, 37, 39-41, 44, 45, 47]. Notably, five patients had neither LC nor NBTE diagnosed while alive and were discovered concurrently during autopsy [18, 21, 26, 31, 38].

### 3.8 Metastasis

The site of metastatic spread was described in 24 of the 32 patients. A total of 41 organ systems were involved among these 24 patients. The most common site of metastasis included lymph nodes 39.0% (n=16) [16, 23, 25-27, 29-31, 33, 39-41, 43, 44, 47], liver 17.1% (n=7) [16, 22, 32, 40, 45-47], bone 14.6% (n=6) [22, 29, 32, 40, 44, 46], brain 14.6% (n=4) [36, 44, 47], adrenal gland 7.3% (n=3) [36, 39, 44], kidney 4.9% (n=2) [22, 39], spleen 2.4% (n=1) [32], muscle 2.4% (n=1) [39], and small bowel 2.4% (n=1) [18].

Cancer stage was specified in 22 of the 32 patients. Most patients were stage IV 86.4% (n=21) [16-18, 22, 28, 29, 32, 36, 37, 39, 40, 42], followed by stage III 9.1% (n=2) [31, 41], and stage I 4.5% (n=1) [33].

### 3.9 Treatment

Half of the patients were treated with anticoagulation (n=15/30 specified cases). Of these, 46.7% (n=7) were treated with unfractionated heparin infusion [16, 21, 31, 34, 36, 40, 49], 33.3% (n=5) were treated with enoxaparin [17, 24, 27, 44, 47], 33.3% (n=5) with rivaroxaban [40-42, 44], and 20.0% (n=3) with warfarin [24, 27, 36]. Six patients in this group received multiple anticoagulants as they were transitioned from parenteral to oral therapy, or vice versa. Among these 6, the ultimate intended anticoagulant was heparin infusion (n=2) [21, 40], warfarin (n=2) [24, 36], and enoxaparin (n=2) [27, 34]. Of the 15 cases that were not treated with anticoagulation, a reason for withholding anticoagulation was provided for 7 cases. These included patient death before treatment could be initiated (n=2) [35, 38], hemorrhage or thrombocytopenia (n=3) [23, 25, 26], low clinical suspicion of anticoagulation being necessary (n=1) [33], and patient refusal (n=1) [46].

Antibiotics were administered in 85.7% (n=18) of the 21 specified cases [22-26, 29, 32-36, 41-44, 46, 47]. Two patients underwent cardiac surgery for valve replacement, and two patients underwent lobectomy [16, 18, 27, 47]. Among 26 specified cases, 30.7% were treated with traditional chemotherapy (n=8). Chemotherapy regimens included carboplatin-etoposide (n=2), carboplatin and gemcitabine (n=1), paclitaxel-etoposide-cisplatin (n=1), carboplatin-vinorelbine (n=1), carboplatin-pemetrexed (n=1), and paclitaxel-etoposide-cisplatin (n=1) [16, 27, 36, 44, 45, 47]. The treatment regimen was not specified for one case (n=1) [24]. Three patients were treated with tyrosine kinase inhibitors: erlotinib (n=2) [17, 44] and Osimertinib (n=1) [47]. One patient was treated with the immune checkpoint inhibitor pembrolizumab [41]. One patient received a combination of chemotherapy + tyrosine kinase inhibitor [47]. Among the 14 patients not treated with any systemic oncologic therapy, reasons cited included patient death before treatment could be initiated (n=8), poor patient functional status (n=2), and patient refusal (n=1). Among 28 specified cases, 21.4% received radiation therapy (n=6) [27, 30, 36, 39, 44, 46].

### 3.10 Summarization of Causes of Death

Mortality rate was 77.3% (n=24) among the specified patients at time of case report publication. The reports described that of these, 8 were most likely a direct consequence of NBTE [16, 21-23, 25, 28, 36, 38, 42]. Causes of death included cerebral infarction (n=3) [21, 23, 28], cerebral hemorrhage (n=2) [25, 42], complete valvular occlusion (n=2) [16, 36], and myocardial infarction (n=1) [38]. The remaining 16 deaths were of unspecified cause.

81.3% (13 of 16) patients with aortic valve involvement were reported dead by the end of the study as well as 75.0% (3 of 4) with multi-valvular disease and 66.7% (8 of 12) with only mitral pathology [16, 18, 21-26, 28-36, 38-43, 45].

Of 7 patients who survived hospitalization, 85.7% (n=6) received anticoagulation during their hospitalization [16, 17, 27, 44, 44, 47]. Of the 24 who died, 41.1% (n=10) received anticoagulation [16, 21, 22, 24, 31, 34, 36, 40-42]. Among the 4 patients who received DOACs, the mortality rate was 75% (n=3) [40-42]. Among 7 patients who received heparin infusion, the mortality rate was 85.7% (n=6) [16, 21, 31, 34, 36, 40]. Among 5 patients treated with enoxaparin, one died, and the other four patients had good clinical outcomes, with two having documentation of resolved vegetations [24, 44, 47]. Both patients in whom lobectomy was performed were treated with anticoagulation and survived [27, 47]. Both patients in whom valve replacement was performed died [16, 18].

Of the 8 patients treated with traditional chemotherapy, 37.5% (n=3) died [24, 36, 45], compared to 90.9% (n=20) of the 22 patients not treated with any systemic chemotherapy. The 3 patients treated with tyrosine kinase inhibitors survived and had favorable clinical outcomes [16, 17, 44]. The patient treated with pembrolizumab and half of the 6 patients treated with radiation died [30, 36, 41, 46].

## 4 DISCUSSION

NBTE is a rare complication of various advanced malignancies, including advanced LC. Case reports comprise the majority of the available published literature. We aimed to identify trends in clinical presentation, management, and outcome of NBTE in patients with LC to elucidate features of this subset of patients.

We found the most common initial presentation was CVA, consistent with the finding that the majority had left-sided valve involvement. Vegetations in NBTE are more fragile and prone to embolize than those from infective (bacterial) endocarditis [49-51]. Patients also experienced splenic and renal thromboembolic events at relatively high rates. Other presentations were nonspecific, such as chest pain, dyspnea, and fever. Prior reports suggested mitral valve involvement is the most common, while we found a predominance of aortic valve involvement [3].

While most patients in this review were diagnosed with LC prior to presentation with NBTE, five were diagnosed with NBTE and LC concurrently or postmortem. While the reports do not provide details about risk factors and cancer screening details for each patient, our findings highlight the possibility of missed opportunities for early diagnosis of LC and appropriate treatment.

Previous literature suggested that NBTE is more commonly associated with advanced malignancy [2, 14, 52]. Interestingly, one patient in this review was reported to have stage I LC. The association of NBTE with advanced disease may be because patients with late-stage disease with multiorgan dysfunction undergo more extensive investigation for NBTE. Adenocarcinoma was the most common subtype of LC associated with NBTE consistent with other observations that adenocarcinoma at other sites is also associated with NBTE [2, 14, 52]. This is unsurprising, given that adenocarcinoma is the most common form of LC [2, 14, 52].

Treatment for NBTE primarily consists of anticoagulation in conjunction with treatment of the underlying cause [9, 13-15, 53]. In this review, only 50% of patients received anticoagulation. Three cases report a contraindication to anticoagulation, such as hemorrhage or thrombocytopenia. In one case, NBTE was discovered only on autopsy. Anticoagulation was not started prior to death because the patient had stage 1 cancer, and there was low clinical suspicion for NBTE. However, given the possibility of more subtle clinical manifestations of NBTE in the early stages of cancer, future investigations should explore the role of screening for NBTE and the potential benefits of early anticoagulation in this often fatal condition. Our findings indicate heterogeneity in the management of NBTE, likely due to a lack of consensus, delayed diagnosis, and absence of evidence-based consensus guidelines in the context of concomitant malignancy. As the pathophysiology for NBTE revolves around a hypercoagulable state, often from an underlying malignancy, anticoagulation is considered an important component of treatment [9, 16]. However, consensus on optimal timing and agents for anticoagulation in NBTE is lacking. In our review, patients treated with enoxaparin had more favorable outcomes than those treated with DOACs unfractionated heparin or warfarin, possibly due to relative clinical stability or socioeconomic factors enabling access to these newer agents. Additional data are needed to inform conclusions regarding the optimal modality of anticoagulation. With the expanding use of DOACs, future studies should investigate their effectiveness relative to traditional agents. Cardiac surgery for NBTE is controversial. Some have applied surgical indications for infective endocarditis to patients with NBTE. Our analysis included two patients with LC and NBTE who underwent cardiac surgery for management, both of whom died [16, 18]. This may reflect more severe disease in patients in whom surgical intervention is attempted, and therefore higher operative risk. The implications of LC on operative risk among patients with NBTE should be further investigated.

The majority of patients in our analysis received no treatment for their LC. This likely reflects the advanced stage and severity of illness at the time of NBTE diagnosis precluded oncologic treatment. The mortality rate for patients treated with chemotherapy (37.5%) was less than that for patients not treated with chemotherapy (90.9%). While most patients in this review did not survive, only eight deaths were attributed directly to NBTE, again underscoring the relatively advanced stage of LC and possibly other comorbidity. Additionally, while most patients who survived were treated with anticoagulation, more severe disease and comorbidity may have precluded anticoagulation in this cohort. Additionally, this review is limited by the heterogenous nature of case reports as not all cases had the same length of follow up to assess mortality rate in patients with LC and NBTE more accurately.

NBTE is a rare disease that affects patients primarily with advanced malignancy. NBTE can occur concurrently with malignancy, with LC being among the most common sites [7]. Patients with LC and NBTE appear to have high mortality, similar to other advanced malignancies with NBTE [7]. NBTE may be more common in LC compared with other malignancies due to several pathophysiologic and clinical factors. For example, adenocarcinoma is strongly associated with a hypercoagulable state, which may be due to overexpression of tissue factors and procoagulant Extracellular Vesicles (EVs). High levels of EVs have been shown to decrease clotting time and are associated with an increased risk of downstream thrombotic events [54, 55]. Furthermore, lung cancer is often diagnosed at an advanced stage, when metastatic spread is already present, which is a known risk factor for venous thromboembolism and NBTE [56-58].

It is possible that NBTE is under-diagnosed since no screening procedures are routinely undertaken in asymptomatic or minimally symptomatic individuals. Some literature may support the use of Pulsed Wave Tissue Doppler Imaging (PW-TDI) for a more accurate assessment of intra-cardiac thrombi and vegetation. For example, in a study that assessed 83 patients with echocardiographically confirmed left ventricular thrombi using PW-TDI, those with an elevated mass peak antegrade velocity were more likely to have cardioembolic events. These measurements can potentially be used as a risk stratification tool to measure the embolic potential of NBTE vegetation [59]. Further research is needed to determine the optimal treatment strategy for patients with LC and NBTE. Enhanced clinician awareness may facilitate earlier diagnosis and improve the effectiveness of treatment.

Our report is limited by the relatively sparse literature on patients with LC-associated NBTE. Our search yielded only case reports and series, with a small total number of reported cases. To address this limitation, we used MMAT to assess the quality of the reports included. All 31 reports generated by our search query had clear descriptions of patient demographics and clinical outcomes, and all had a low risk of nonresponse bias, thus, the studies scored very highly on MMAT. While we acknowledge the valuable insights provided by these case reports, we recommend caution in interpreting the results as being broadly applicable. This is a reflection of the limited available knowledge on lung cancer associated with NBTE and shows the potential of this area as a future research focus.

## CONCLUSION

Examining the literature, it is evident that there is a significant prevalence of adverse outcomes in patients with NBTE and LC. As such, clinicians should have a high suspicion for NBTE in LC patients presenting with embolic manifestations. Prompt recognition of NBTE in this population can lead to treatment changes that can prevent adverse clinical events. Though our study identified smoking and adenocarcinoma as NBTE risk factors, in the future, larger studies are needed to identify LC patients at high risk for NBTE development and better treatments for LC-related NBTE.

## Figures and Tables

**Table 1:** Patient characteristics.

**Author**	**Patient**	**Presenting Diagnosis**	**Organ** **Infarcts**	**Metastasis**	**Initial** **Diagnosis (NBTE or LC)**	**Time to** **Diagnosis (NBTE or LC)**	**Valves** **Affected**	**Treatment** **(Anticoagulation)**	**Outcome**	**Cause of Death**
Zhou *et al.*	69M	Pneumonia, CVA	Brain, Eye	Liver, Spleen, Bone	NBTE	1 day	Mitral	No	Deceased	LC-lung collapse
Gray *et al.*	66M	Pneumonia	Heart	Liver	LC	10 days	Mitral	No	Deceased	Unspecified
Umeojiako *et al.*	44M	NBTE	None	Liver, Bone	NBTE	Unspecified	Mitral	No	Alive	N/A
Clough and Duncan	65F	Psychosis	Spleen, Lung, Brain, Kidney	Lymph nodes	LC	Unspecified	Aortic	None due to thrombocytopenia	Deceased	NBTE - brain infarcts
Nagao *et al.*	87M	DIC	Kidney	Mediastinal lymph nodes	LC	2 years	Tricuspid and Mitral	No	Deceased	Unspecified
Palicherla *et al.*	71M	CVA	Brain	Unspecified	LC	Unspecified	Aortic	Unspecified	Unspecified	Unspecified
Mitma *et al.*	68M	LC	Brain	Unspecified	LC	Same admission	Mitral	Enoxaparin	Alive	N/A
Ferreira *et al.*	67F	PE	Lung, Brain	Unspecified	NBTE	7 days	Aortic	No - died before could be initiated	Deceased	LC - respiratory failure
Studdy *et al.*	40M	DVT/PE	Heart, Lung, Kidney	Unspecified	Both discovered on autopsy	Same time - discovered on autopsy	Mitral	No - died before could be initiated	Deceased	NBTE - cardiac thrombosis
Royter and Cohen	68M	CVA; PE	Brain, Heart	Unspecified	LC	2 weeks	Mitral	Enoxaparin initially then warfarin	Deceased	Unspecified
Tamura *et al.*	62F	NBTE	Kidney, Brain, Spleen	Left lung	NBTE	30 days	Aortic	Rivaroxaban	Deceased	NBTE-cerebral hemorrhage with midline shift
Shibata *et al.*	67F	CVA	Brain, Kidney, Spleen, Heart	Lymph nodes	NBTE	Unspecified	Mitral	No; had hemorrhagic stroke	Deceased	NBTE - cerebral hemorrhage
Lee *et al.*	60F	Cardiac arrest	Kidney, Spleen, Brain	Lymph nodes	Discovered on autopsy	Unspecified	Mitral	No; had cardiac arrest	Deceased	Unspecified
Wong *et al.*	34F	CVA	Spleen, Brain	Lymph nodes	NBTE	Unspecified	Aortic	Warfarin initially then enoxaparin	Alive	N/A
Yoshii *et al.*	63F	DIC	Brain, Lung	Lymph nodes	LC	Unspecified	Mitral	Heparin	Alive	N/A
Tai *et al.*	68F	CVA	Brain	Unspecified	LC	21 days	Aortic	No	Deceased	NBTE-MCA infarct
Neilan *et al.*	57M	Pneumonia	Brain, Lung, Spleen, Kidney	Lymph nodes	NBTE	Unspecified	Mitral	No	Deceased	Unspecified
Chisholm *et al.*	55F	LC	Spleen, Brain, Kidney	Lymph nodes, Muscle, Adrenals, Kidney, Renal vein	LC	Unspecified	Aortic	No	Deceased	Unspecified
Kooiker *et al.*	58M	CVA	Brain	Small bowel	Both discovered on autopsy	Same time - discovered on autopsy	Aortic	No	Deceased	Unspecified
Khan *et al.*	55M	CVA	Brain	Liver	NBTE	Unspecified	Mitral initially, then aortic and tricuspid	Heparin	Deceased	NBTE -valve closure secondary to multiple valvular infarcts
Wigger *et al.*	66M	CVA	Brain	Lymph nodes, Bone	NBTE	4 days	Aortic	No	Deceased	Unspecified
Olney *et al.*	47F	CVA	Brain	Lymph nodes	LC	1 month	Aortic	No	Deceased	Unspecified
Kim *et al.*	72F	PE	Spleen, Brain	Liver, Brain, Lymph nodes	LC	11 months	Mitral	Enoxaparin	Alive	N/A
Xie *et al.*	59M	Pericardial effusion	None	Lymph nodes, Bone, Brain	LC	2 months	Aortic	Enoxaparin	Alive	N/A
Xie *et al.* (second patient)	55F	LC	None	Lymph nodes, Adrenal, Brain	LC	18 months	Aortic and Mitral	Rivaroxaban	Alive	N/A
Taniyama *et al.*	37M	LC	None	Adrenal, Brain	LC	4 months	Aortic	Heparin initially; bridged to warfarin	Deceased	NBTE - complete occlusion of aortic valve from thrombus
Sand *et al.*	44M	CVA	Heart, Kidney, Lung, Spleen	Lymph nodes	Both discovered on autopsy	Same time - discovered on autopsy	Aortic	Heparin	Deceased	Unspecified
Gunderson and Moynihan	59M	CVA	Brain. Spleen, Kidney	Unspecified	Both discovered on autopsy	4 days	Aortic and Mitral	Was already on rivaroxaban for DVT/PE 2 weeks prior. Heparin while admitted.	Deceased	NBTE- MCA syndrome
Cheung *et al.*	44F	CVA	Brain, Spleen	Unspecified	LC	5 days	Aortic	Heparin initially then enoxaparin	Deceased	Unspecified
Rua *et al.*	65F	PE	Heart	Liver, Bone, Kidney	NBTE	5 days	Aortic	Yes-unspecified which type	Deceased	NBTE
Panicucci *et al.*	64F	DVT/PE, CVA	Brain	Lymph nodes, Bone, Liver	LC	Unspecified	Aortic	Initially on rivaroxaban for DVT/PE transitioned to heparin after NBTE discovered	Deceased	LC - cachexia and progressive neurologic symptoms
Perrone *et al.*	63M	PE, CVA	Spleen, Kidney, Brain, Bowel	Lymph nodes	LC	Over 5 days	Mitral	Rivaroxaban	Deceased	Unspecified

**Abbreviations:** F: Female, M: Male.

**Table 2:** Diagnostic workup.

**Author**	**Patient**	**Blood Culture**	**Echocardiogram**	**CT Chest Findings**
Zhou *et al.*	69M	Negative	6 mm x 2mm vegetation on anterior mitral leaflet with mild MR; no WMA; EF 62%	Left upper lobe malignancy. possible lymphangitic carcinomatosis. consolidation in the left upper lobe, middle lobe and left lower lobe likely related to tumor infiltration. Metastatic lesions in liver, spleen, bone
Gray *et al.*	66M	Negative	Thickening of mitral leaflets with multiple small valvular vegetations; severe MR	Right hilar mass with lymphangitic spread to middle and lower lobe; obstructive lobar pneumonia; hepatic metastases
Umeojiako *et al.*	44M	Negative	Normal LV systolic function, echogenic mobile structure on posterior leaflet of mitral valve	Multiple cavitating and subpleural lesions, widespread liver lesions, lytic lesions in spine with para-aortic adenopathy
Clough and Duncan	65F	Negative	Thickening of aortic valve with vegetation	Chest CT normal
Nagao *et al.*	87M	Negative	Apical LV aneurysm without thrombus; tricuspid valve with isoechoic vegetation with severe TR; thickened mitral valve without vegetation	CT with spiculated mass in left upper lung lobe
Palicherla *et al.*	71M	Negative	Thickening of aortic valve leaflet tips with moderate AR	Unspecified
Mitma *et al.*	68M	Negative	Echodensity on anterior mitral valve leaflet; mild MR; no LV thrombus	Right upper lobe mass
Ferreira *et al.*	67F	Negative	Aortic vegetation with moderate to severe AR	CT with large right pleural effusion, pleural nodules
Studdy *et al.*	40M	Negative	Not performed - discovered on autopsy	Unspecified
Royter and Cohen	68M	Negative	Large complex mass on anterior mitral leaflet, 1 cm section of mass was mobile; severe MR; R-L shunt at level of PFO, normal LV systolic function	Unspecified
Tamura *et al.*	62F	Negative	Moderate AR; vegetations on non-coronary and left coronary cusps of aortic valve	CT with 10-mm nodular lesion with pleural indentation in R lung; unchanged from prior 4 years
Shibata *et al.*	67F	Negative	Vegetation on anterior and posterior mitral leaflet; moderate MR	Left inferior lobe cancer with multiple mediastinal lymph node metastases
Lee *et al.*	60F	Unspecified	Not performed - discovered on autopsy	Opacity in left lower lobe
Wong *et al.*	34F	Negative	Mildly thickened aortic valve; mass attached to right coronary cusp of aortic valve	Left hilar mass
Yoshii *et al.*	63F	Negative	Vegetation on anterior and posterior mitral leaflet	2.5 cm nodule attached to hilar lymph nodes in left lower lobe and lymphadenopathy in mediastinum and left axilla
Tai *et al.*	68F	Negative	7 cm x 6 cm vegetation on non-coronary cusp of aortic valve	Unspecified
Neilan *et al.*	57M	Negative	TTE with LVEF 35%, diffuse hypokinesis; no PFO; mitral leaflets focally thickened, mild-moderate MR; small circumferential pericardial effusion. TEE with diffuse echogenic material on left atrial surface and anterior and posterior mitral leaflets	Consolidation of left lower lobe, progressed from prior
Chisholm *et al.*	55F	Negative	Concentric LV hypertrophy with decreased LV compliance and calcification of mitral annulus	5 cm irregular left hilar mass and 1.1 cm nodular lesion in left upper lobe
Kooiker *et al.*	58M	Unspecified	Not performed - discovered on autopsy	Not performed
Khan *et al.*	55M	Negative	1 cm vegetation on posterior mitral valve leaflet with thickened anterior leaflet: moderate MR	Right upper lobe lesion with liver metastases
Wigger *et al.*	66M	Negative	6 x 6mm mobile structure on R coronary cusp of aortic valve with moderate AR	Consolidation in left lower lobe with enlarged mediastinal lymph nodes
Olney *et al.*	47F	Negative	Not performed	R hilar mass with supraclavicular lymphadenopathy
Kim *et al.*	72F	Negative	Thickening of mitral leaflets with severe MR; two masses of mitral valve with leaflet destruction	Right upper lobe lesion with enlarged lymph nodes; liver metastases
Xie *et al.*	59M	Negative	LVEF decreased from 73% to 51%; large pericardial effusion; mobile mass 22 x 21 mm on anterior wall of ascending aorta	Right lung carcinoma with multiple bony metastases
Xie *et al.* (second patient)	55F	Negative	LVEF 58%; multiple thrombi on aortic and mitral valve	Cancer in basal segment of right lower lobe with hilar and multiple mediastinal lymph node metastases
Taniyama *et al.*	37M	Unspecified	Not performed - discovered on autopsy	Irregular mass in left lung apex with diameter 55 mm and PE
Sand *et al.*	44M	Negative	Echodensity near aortic valve and anterior leaflet of mitral valve	Not performed
Gunderson and Moynihan	59M	Unspecified	Not performed - discovered on autopsy	Unremarkable
Cheung *et al.*	44F	Negative	Moderate AR; mobile vegetation on right coronary cusp of aortic valve	4.6 cm thick-walled cavitary lesion in left lower lobe
Rua *et al.*	65F	Negative	Initial TTE negative. TEE with 8 mm vegetation on aortic valve between coronary and noncoronary cusp	Moderate left pleural effusion, consolidation of left lower lobe, opacities/GGOs in left lung. Adenopathy in mediastinum and hilum.
Panicucci *et al.*	64F	Negative	Severe AR; thickened aortic valve with possible vegetation; no PFO	Pulmonary nodule with metastases to liver and bone
Perrone *et al.*	63M	Negative	Mobile echogenic mass 0.8 x 0.6 cm on atrial surface of anterior leaflet of mitral valve; moderate AR; reduced LVEF of 40%; no PFO	PE; mass in right lower lobe; ipsilateral mediastinal lymph node involvement

**Abbreviations:** F: Female, M: Male.

## Data Availability

The data and supportive information are available within the article.

## LIST OF ABBREVIATIONS

A/P: Abdomen and Pelvis
APLS: Antiphospholipid Syndrome
AR: Aortic Regurgitation
AS: Aortic Stenosis
AV: Aortic Valve
CT: Computed Tomography
CVA: Cerebrovascular Accident
DIC: Disseminated Intravascular Coagulation
DOAC: Direct Oral Anticoagulants
DVT: Deep Vein Thrombosis
EF: Ejection Fraction
LC: Lung Cancer
LV: Left Ventricle
MI: Myocardial Infarction
MR: Mitral Regurgitation
MV: Mitral Valve
NBTE: Non-bacterial Thrombotic Endocarditis
PE: Pulmonary Embolism
RA: Right Atrium
SLE: Systemic Lupus Erythematosus
TEE: Transesophageal Echocardiography
TIA: Transient Ischemic Attack
TTE: Transthoracic Echocardiography
TV: Tricuspid Valve

## STANDARDS OF REPORTING

PRISMA guidelines and methodology were followed.

## SUPPLEMENTARY MATERIAL

PRISMA checklist is available as supplementary material on the publisher’s website along with the published article.
